# Thyroid dermopathy—a diagnostic clue of hidden hyperthyroidism

**DOI:** 10.4161/19381980.2014.981078

**Published:** 2015-01-26

**Authors:** Tapan Kumar Dhali, Monica Chahar

**Affiliations:** Department of Dermatology; Employees' State Insurance Post Graduate Institute of Medical Science and Research; New Delhi, India

**Keywords:** Graves’ disease, hyperthyroidism, mucin, pretibial myxedema, thyroid dermopathy

## Abstract

Thyroid dermopathy is an uncommon manifestation of autoimmune thyroid disease. About 0.5%–4.3% of patients with history of thyrotoxicosis and 15% of patients with severe Graves’ ophthalmopathy have this cutaneous manifestation. However thyroid dermopathy is almost always associated with ophthalmopathy (96%) and sign and symptoms of hyperth-yroidism. The diagnosis of thyroid dermopathy is based on clinical sign and symptoms, serological thyroid hormone abnormalities supported by skin pathology. Isolated dermopathy is an uncommon manifestation of hyperthyroidism. A 35-year-old male presented with 7 months history of asymptomatic, multiple skin colored nodulo-tumorous growth over anterior aspect of both leg and one erythematous plaque with mild central atrophy on left leg. On examination most of the nodulo-tumorous growth (1 cm × 1 cm to 4 cm × 4 cm) and plaque (3 cm × 4 cm) showed ‘peau d’ orange’ appearance and were firm in consistency, indurated, non-tender with no rise of local temperature. Complete systemic and ophthalmological examination revealed no abnormalities. Abnormal thyroid function test and cutaneous deposition of mucin on histopathology suggested the diagnosis.The case is reported for its uncommon manifestation. Clinical sign should be documented and analysis of skin histopathology should be carried out in patients with suspected thyroid dermopathy.

## Introduction

Thyroid dermopathy is an infrequent manifestation of autoimmune thyroid disease characterized by localized thickening of the skin commonly seen in the pretibial area. It is almost always associated with ophthalmopathy (96%) and sign and symptoms of hyperthyroidism. The diagnosis of thyroid dermopathy is based on clinical sign and symptoms, serological thyroid hormone abnormalities (↑T3, ↑T4 and ↓TSH) supported by skin pathology. Isolated dermopathy is a rare manifestation of hyperthyroidism. We report a case in whom cutaneous myxedema was the initial manifestation of hyperthyroidism, leading to the diagnosis of Graves’ disease.

**Figure 1.**
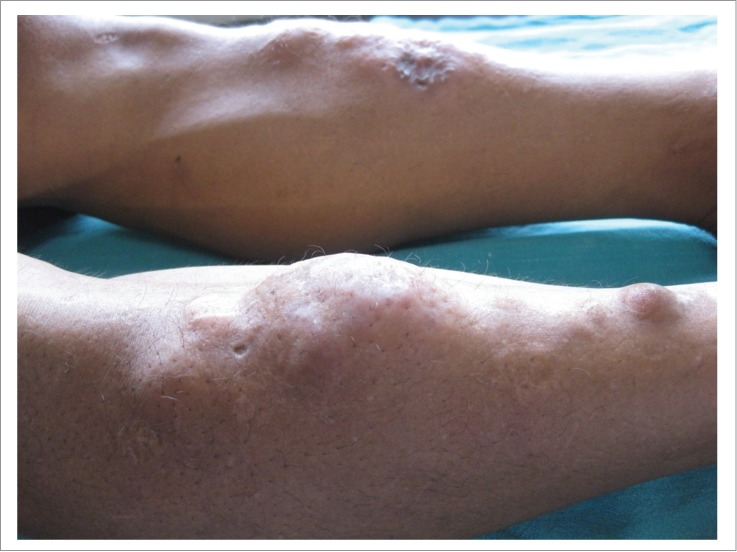
Nodules and plaque with central atrophy over right shin.

**Figure 2.**
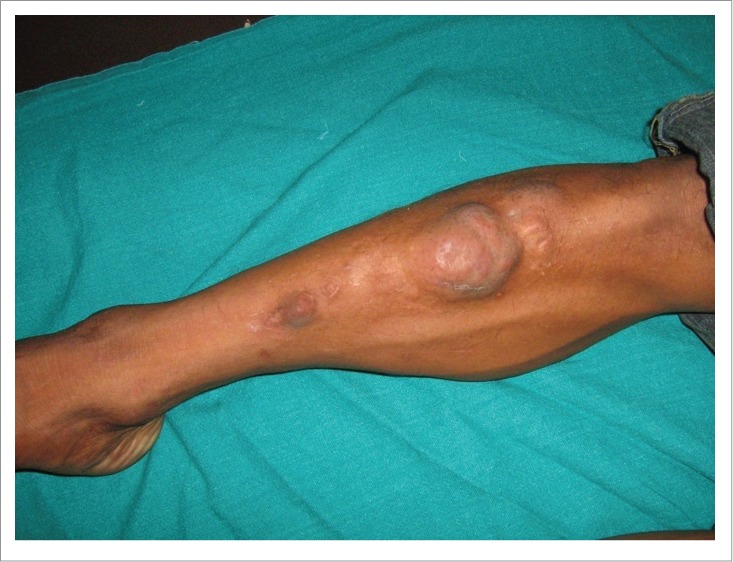
Nodules and tumors on right leg at the presentation.

**Figure 3.**
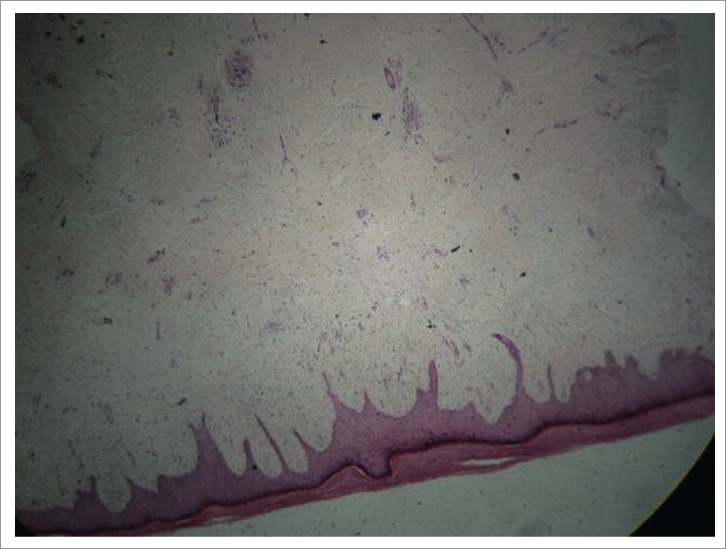
Dermal infiltrate of mucin with separation of collagen bundles: Haematoxylin and eosin; original magnification, 4X.

**Figure 4.**
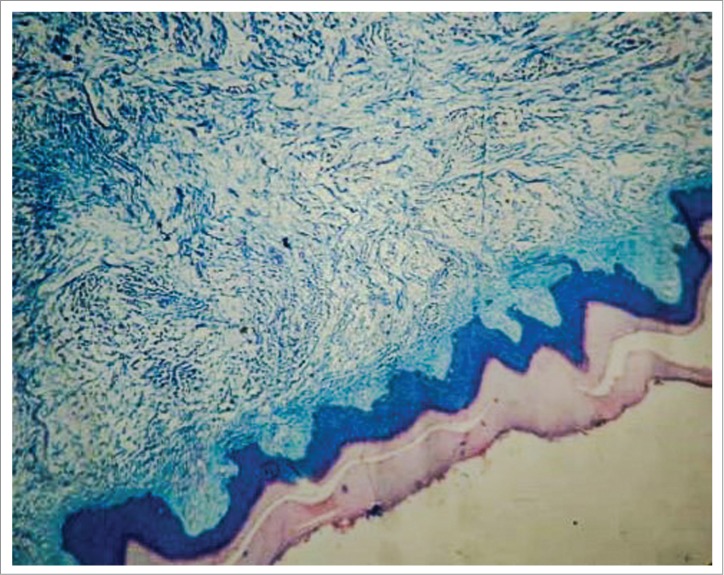
Deposition of mucin in the dermis causing wide separation of the collagen bundles: Alcian Blue stain; 4X.

**Figure 5.**
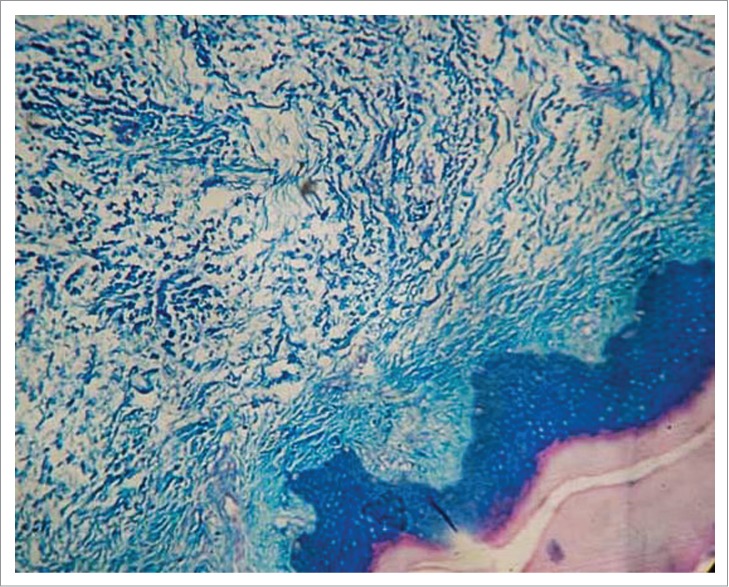
Infiltration by mucin: Alcian Blue stain; 10X.

**Figure 6.**
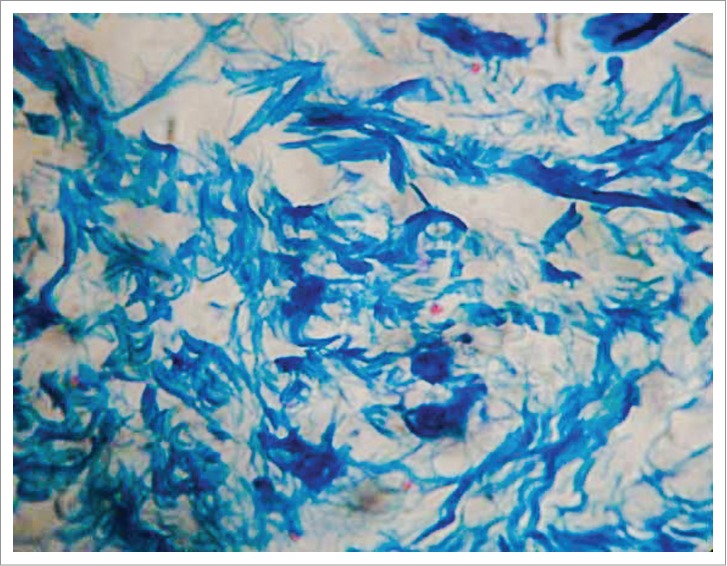
Demonstration of mucin with Alcian Blue stain; 40X.

## Case Description

A 35-year-old male presented with 7 months history of asymptomatic, multiple skin colored tumorous growths over anterior aspect of both legs along with the presence of a gradually progressive reddish raised lesion on his left lower leg.

Cutaneous examination revealed presence of multiple skin colored nodules over the extensor aspect of bilateral shins ranging in size from 1 cm × 1 cm to 4 cm × 4 cm. There was a single erythematous plaque of size 3 cm × 4 cm present over left shin having mild central atrophy. Most of the nodules and plaque showed dilated hair follicle openings giving a ‘peau d’ orange’ appearance (**Fig. 1 ****and 2**).

On palpation they were firm in consistency, indurated, non-tender and surface temperature was normal. Rest of the systemic examination was within normal limits.

With a provisional diagnosis of pretibial myxedema, the patient was investigated. His routine blood investigations, chest X-ray and ECG were within normal limits. Two punch biopsies were taken, one from a nodule and the second from the margin of the erythematous plaque.

Histopathological examination of lesional skin on alcian blue staining revealed dermal mucin deposition, particularly in the lower dermis causing wide separation of collagen bundles (**Fig. 3 –6**).

Thyroid Function Test showed- T3-11.0 ng/ml (0.69–2.02 ng/ml); T4-17.2 g/dl (4.4–11.6 g/dl); TSH- 0.6 mIU/L (0.4–6.2 mIU/L). Anti-TSH receptor antibody titres were elevated as well. In contrast, anti-TPO and antithyroglobulin antibodies were negative. Radioactive iodine uptake was elevated in a diffuse pattern.

Based on laboratory and histopathological reports, a diagnosis of hyperthyroidism due to Grave's disease with cutaneous myxedema was made and the patient was sent to medicine and ophthalmology departments for complete evaluation. Ophthalmologic evaluation was negative for Graves’ ophthalmopathy. Apart from mild heat intolerance, no other features of hyperthyroidism were found.

The patient was started on oral carbimazole tablet (15 mg OD) along with intralesional triamcinolone 40 mg/ml in every 3 weeks. By the end of 3 months, almost complete flattening of the lesions was seen.

## Discussion

Thyroid dermopathy or localized myxedema is characterized by localized thickening of the skin and is a late and rare manifestation of autoimmune thyroiditis, particularly of Graves’ disease.^1^ About 0.5%–4.3% of patients with history of thyrotoxicosis and 15% of patients with severe Graves’ ophthalmopathy have this cutaneous manifestation.^2,3^ It most commonly affects middle-aged females with female to male ratio of approximately 4: 1.^4^ It is commonly localized in the pretibial area and is therefore often referred to as pretibial myxedema.^1^

Almost 97% of dermopathy patients have coexisting ophthalmopathy and features of hyperthyroidism.^5^ Generally, thyrotoxicosis develops first, followed by ophthalmopathy and finally dermopathy in patients who have all of these manifestations.^2,6^ Cutaneous myxedema in the absence of, or preceding ophthalmopathy, or as the initial manifestation of hyperthyroidism, is rare.

Several theories have been put forward to explain the exact pathogenesis of PM. Autoantibodies against thyroid antigens and reactive T lymphocytes are thought to cross-react with connective tissue and muscle antigens. TSH receptor antibodies binding to the receptors in the connective tissue may stimulate fibroblasts to produce a large amount of glycosaminoglycans.^2^ The polymerase chain reaction has demonstrated ribonucleic acid encoding the extracellular domain of the TSH receptor in cultured orbital, abdominal skin, and peripheral skin fibroblasts from patients with ophthalmopathy or localized myxedema, and also in skin from normal subjects.^7,8^

It was speculated that pretibial fibroblast may react with T cell lymphocytes on their thyrotropin receptors and then they may overproduce glycosaminoglycans.^9^ Stimulation of fibroblast by TSH-receptor antibodies along with mechanical factors and venous stasis causes accumulation of mucin.^4,9^ There is evidence that trauma and injury may lead to the activation of T cells and the initiation of an antigen specific response, in this case the activation of fibroblasts and production of GAGs.^10^

Most common clinical presentation is in the form of nonpitting edema and induration of the skin giving a ‘peau d’orange’ appearance along with occasional raised, hyperpigmented, violaceous papules. Other clinical variants of thyroid dermopathy are plaques, nodules, and had polypoid or elephantiasic type lesions.^2^ It is usually asymptomatic; however, hyperhidrosis limited to the affected region has been described.^11^ Quantitative measurement of stimulated sweat after intradermal injection of methacholine shows that sweating is 2–4 times greater in lesional skin than in perilesional skin. The latter may be due to stimulation of sympathetic fibers by the surrounding deposition of mucin.^12,13^ A case of reversible foot drop due to entrapment neuropathy has been reported.^14^ The elephantiasic form of pretibial myxedema is the most symptomatic form and creates mechanical and functional disability. Patients with this condition are prone to all the complications and morbidity seen in lymphedema.^15^

Localization of lesions commonly occurs in Pretibial (99.4%), pretibial+ feet (4.3%), pretibial + upper extremities (1.1%), preradial aspect of the arms, upper back, shoulders, pinnae, nose, thigh and toes. The predilection of localization to the pretibial area may result because of local trauma with additional mechanical (gravitational forces) or anatomic (site-specific differences in fibroblasts) factors. ^5^

Histopathological examination of cutaneous myxedema reveals typical mucin deposition and separation of normal collagen bundles by mucin when the tissue is stained with alcian blue and the periodic acid- Schiff. Also, compared with normal skin, the number of collagen fibers is relatively reduced, and there is marked edema. Occasionally, hyperkeratosis, acanthosis, and papillomatosis are noted.^16^ Ultrasonography **(**10-MHz) has been used to document the increased thickness of pretibial myxedema.^5^

Treatment of cutaneous myxedema is often challenging. Control of thyrotoxicosis has been shown to have no effect on skin lesions. Intralesional or topical corticosteroids with or without occlusion, complete decompressive physiotherapy, surgical excision have been tried with good response in mild cases.^2^

Newer therapies include octreotide (somatostatin analog), an insulin analog (↓TSH-receptor insulin-like growth factor-1activity), and pentoxifylline, which decreases glycosaminoglycans by fibroblasts have been tried.^17^ High-dose IV Immunoglobulin treatment^18^ and plasmapheresis^19,20^ have also been used to treat PTM in a few patients and have led to improvement or remission of the condition.

The long-term outcome and natural course of treated and untreated localized myxedema have been reported in a series of 178 patients.^5^ Out of these patients, 46% did not require any therapy. In mild cases that did not require any treatment, 50% of the patients had complete remission within 17 years; 70% of milder untreated cases and 58% of severe cases treated with local therapy had either a partial or complete remission.^5^

## Conclusion

Isolated lesions of the thyroid dermopathy in the absence of ophthalmopathy or other evidence of hypert-hyroidism is a rare presentation and represents a diagnostic challenge.
